# Supplementary material for: Pyruvate Dehydrogenase Kinase 1 inhibition mediated oxidative phosphorylation enhancement in cartilage promotes osteoarthritis progression
Source: BMC Musculoskelet Disord. 2023 Jul 20;24:597. doi: 10.1186/s12891-023-06585-6 (PMC10357736; doi:10.1186/s12891-023-06585-6)
Supplement: Supplementary file 1 — Additional file 1. [file 12891_2023_6585_MOESM1_ESM.zip › KEGG.Gsea.1653622432464/KEGG_PROXIMAL_TUBULE_BICARBONATE_RECLAMATION.html]

Details for gene set KEGG\_PROXIMAL\_TUBULE\_BICARBONATE\_RECLAMATION[GSEA]

|  || Dataset | 55457\_collapsed\_to\_symbols.cls.cls #OA\_versus\_normal.cls.cls #OA\_versus\_normal\_repos |
| Phenotype | cls.cls#OA\_versus\_normal\_repos |
| Upregulated in class | OA |
| GeneSet | KEGG\_PROXIMAL\_TUBULE\_BICARBONATE\_RECLAMATION |
| Enrichment Score (ES) | 0.27888507 |
| Normalized Enrichment Score (NES) | 0.9401871 |
| Nominal p-value | 0.52403843 |
| FDR q-value | 0.7409665 |
| FWER p-Value | 1.0 |
Table: GSEA Results Summary

  

Fig 1: Enrichment plot: KEGG\_PROXIMAL\_TUBULE\_BICARBONATE\_RECLAMATION      
 Profile of the Running ES Score & Positions of GeneSet Members on the Rank Ordered List

  

| SYMBOL | TITLE | RANK IN GENE LIST | RANK METRIC SCORE | RUNNING ES | CORE ENRICHMENT || 1 | MDH1 | malate dehydrogenase 1 [Source:HGNC Symbol;Acc:HGNC:6970] | 271 | 0.772 | 0.0914 | Yes |
| 2 | PCK2 | "phosphoenolpyruvate carboxykinase 2, mitochondrial [Source:HGNC Symbol;Acc:HGNC:8725]" | 759 | 0.579 | 0.1379 | Yes |
| 3 | ATP1B1 | ATPase Na+/K+ transporting subunit beta 1 [Source:HGNC Symbol;Acc:HGNC:804] | 1177 | 0.481 | 0.1755 | Yes |
| 4 | GLUD1 | glutamate dehydrogenase 1 [Source:HGNC Symbol;Acc:HGNC:4335] | 1279 | 0.462 | 0.2349 | Yes |
| 5 | GLUD2 | glutamate dehydrogenase 2 [Source:HGNC Symbol;Acc:HGNC:4336] | 1554 | 0.420 | 0.2748 | Yes |
| 6 | SLC9A3 | solute carrier family 9 member A3 [Source:HGNC Symbol;Acc:HGNC:11073] | 2148 | 0.344 | 0.2789 | Yes |
| 7 | AQP1 | aquaporin 1 (Colton blood group) [Source:HGNC Symbol;Acc:HGNC:633] | 3225 | 0.228 | 0.2285 | No |
| 8 | FXYD2 | FXYD domain containing ion transport regulator 2 [Source:HGNC Symbol;Acc:HGNC:4026] | 4073 | 0.149 | 0.1844 | No |
| 9 | SLC4A4 | solute carrier family 4 member 4 [Source:HGNC Symbol;Acc:HGNC:11030] | 5027 | 0.071 | 0.1207 | No |
| 10 | ATP1A3 | ATPase Na+/K+ transporting subunit alpha 3 [Source:HGNC Symbol;Acc:HGNC:801] | 5475 | 0.035 | 0.0910 | No |
| 11 | ATP1B4 | ATPase Na+/K+ transporting family member beta 4 [Source:HGNC Symbol;Acc:HGNC:808] | 7306 | -0.103 | -0.0361 | No |
| 12 | CA2 | carbonic anhydrase 2 [Source:HGNC Symbol;Acc:HGNC:1373] | 7708 | -0.135 | -0.0476 | No |
| 13 | ATP1A2 | ATPase Na+/K+ transporting subunit alpha 2 [Source:HGNC Symbol;Acc:HGNC:800] | 8218 | -0.173 | -0.0620 | No |
| 14 | ATP1A1 | ATPase Na+/K+ transporting subunit alpha 1 [Source:HGNC Symbol;Acc:HGNC:799] | 8282 | -0.177 | -0.0411 | No |
| 15 | GLS | glutaminase [Source:HGNC Symbol;Acc:HGNC:4331] | 8423 | -0.187 | -0.0247 | No |
| 16 | SLC25A10 | solute carrier family 25 member 10 [Source:HGNC Symbol;Acc:HGNC:10980] | 8552 | -0.198 | -0.0058 | No |
| 17 | PCK1 | phosphoenolpyruvate carboxykinase 1 [Source:HGNC Symbol;Acc:HGNC:8724] | 9100 | -0.239 | -0.0135 | No |
| 18 | SLC38A3 | solute carrier family 38 member 3 [Source:HGNC Symbol;Acc:HGNC:18044] | 10443 | -0.366 | -0.0643 | No |
| 19 | CA4 | carbonic anhydrase 4 [Source:HGNC Symbol;Acc:HGNC:1375] | 10464 | -0.369 | -0.0121 | No |
| 20 | ATP1B2 | ATPase Na+/K+ transporting subunit beta 2 [Source:HGNC Symbol;Acc:HGNC:805] | 10595 | -0.383 | 0.0336 | No |
| 21 | ATP1B3 | ATPase Na+/K+ transporting subunit beta 3 [Source:HGNC Symbol;Acc:HGNC:806] | 11424 | -0.490 | 0.0407 | No |
| 22 | GLS2 | glutaminase 2 [Source:HGNC Symbol;Acc:HGNC:29570] | 11547 | -0.507 | 0.1051 | No |
Table: GSEA details [plain text format]

  

Fig 2: KEGG\_PROXIMAL\_TUBULE\_BICARBONATE\_RECLAMATION      
 Blue-Pink O' Gram in the Space of the Analyzed GeneSet

  

Fig 3: KEGG\_PROXIMAL\_TUBULE\_BICARBONATE\_RECLAMATION: Random ES distribution      
 Gene set null distribution of ES for **KEGG\_PROXIMAL\_TUBULE\_BICARBONATE\_RECLAMATION**
